# Decoding Hypotension: Porcelain Aorta and Its Unexpected Normalcy

**DOI:** 10.7759/cureus.78417

**Published:** 2025-02-03

**Authors:** Raya Tcheroyan, Jachrise Sibblis, Jesse Liou, Thomas Sewatsky

**Affiliations:** 1 Internal Medicine, Cooper University Hospital, Camden, USA; 2 Hospital Medicine, Cooper University Hospital, Camden, USA; 3 Pulmonary and Critical Care Medicine, Cooper University Hospital, Camden, USA; 4 Critical Care Medicine, Cooper University Hospital, Camden, USA

**Keywords:** blood pressure reading, critical care, general internal medicine, porcelain aorta, shock

## Abstract

Peripheral blood pressure (PBP) is used as a substitute for central blood pressure (CBP) because it is more accessible and less invasive, but in certain conditions like porcelain aorta, it may fail to accurately represent true hemodynamics. Porcelain aorta, characterized by extensive calcification of the aortic arch, can lead to significant discrepancies between PBP and CBP leading to challenges in diagnoses and management. We report a case of an 80-year-old male individual with porcelain aorta, found to have a greater than 100 mmHg difference in systolic PBP and CBP, who presented with recurrent admissions for hypotension despite an absence of organ dysfunction. This case highlights the need to be aware and account for CBP measurements in patients with extensive vascular calcifications to guide appropriate diagnostic and therapeutic decisions.

## Introduction

Peripheral blood pressure (PBP) is used in clinical practice as a substitute for central blood pressure (CBP) due to its accessibility and ease of measurement [1]. Accurate blood pressure measurement is crucial in decision-making, especially in critically ill patients to evaluate hemodynamic stability and help guide management. However, PBP does not always accurately reflect CBP in conditions associated with altered vascular compliance [1]. 

Porcelain aorta is a rare condition characterized by extensive calcification of the ascending aorta and/or aortic arch, often forming a complete or near-complete circumferential ring [2]. This results in significant loss of aortic compliance and elasticity, increasing resistance to blood flow and lowering PBP. Although some studies describe discrepancies between CBP and PBP, they were not as extensive as those observed in our patient [1]. We report a case of an 80-year-old male individual with porcelain aorta, in whom a >100 mmHg difference between PBP and CBP was observed. This is the first documented case of such an extreme discrepancy, highlighting the need for clinicians to be aware of these conditions and consider CBP measurements in patients with advanced vascular calcifications to optimize diagnostic accuracy and therapeutic decisions.

## Case presentation

An 80-year-old male individual, with a relevant history of chronic obstructive pulmonary disease (COPD), coronary artery disease with subsequent percutaneous intervention, tobacco use, and a previous carotid endarterectomy was referred to the hospital from an outpatient clinic after being found hypotensive. He initially presented with complaints of chest pain and increasing dyspnea over the past few weeks. A transthoracic echocardiogram was performed which showed moderate aortic stenosis and apical wall hypokinesis. The patient subsequently underwent a left heart catheterization for further evaluation. Repeated aortic pressures were obtained with the highest at 233/80 mmHg (Table 1). Multiple upper extremity PBP were measured ranging between 112/68 to 152/95 (Table 1). This revealed a significant discrepancy of more than 100mmHg between central and peripheral pressures. He was diagnosed with porcelain aorta due to this marked pressure differential along with severe peripheral vascular disease. In addition, catheterization revealed occluded inferior vena cava (IVC) with collaterals, mild aortic stenosis with a gradient of 10mmHg, and heavily calcified coronaries with total occlusion of the left anterior descending artery due to in-stent restenosis. The patient was treated with intravenous diuretics given concerns of a heart failure exacerbation and was discharged home when medically optimized. 

**Table 1:** Comparison of aortic, left ventricular, and peripheral blood pressure measurements taken during cardiac catherization. SBP: systolic blood pressure, DBP: diastolic blood pressure, MAP: mean arterial pressure, AO: aorta, LV: left ventricle, PBP: peripheral blood pressure.

Site	AO	LV	Blood pressure cuff (PBP)
SBP/DBP (MAP) in mmHg	0	228/26	112/68 (92)
	210/64 (111)	235/25	117/76 (95)
	231/80 (130)	240/8	115/77 (98)
	233/80 (131)	242/10	152/95 (117)

**Figure 1:**
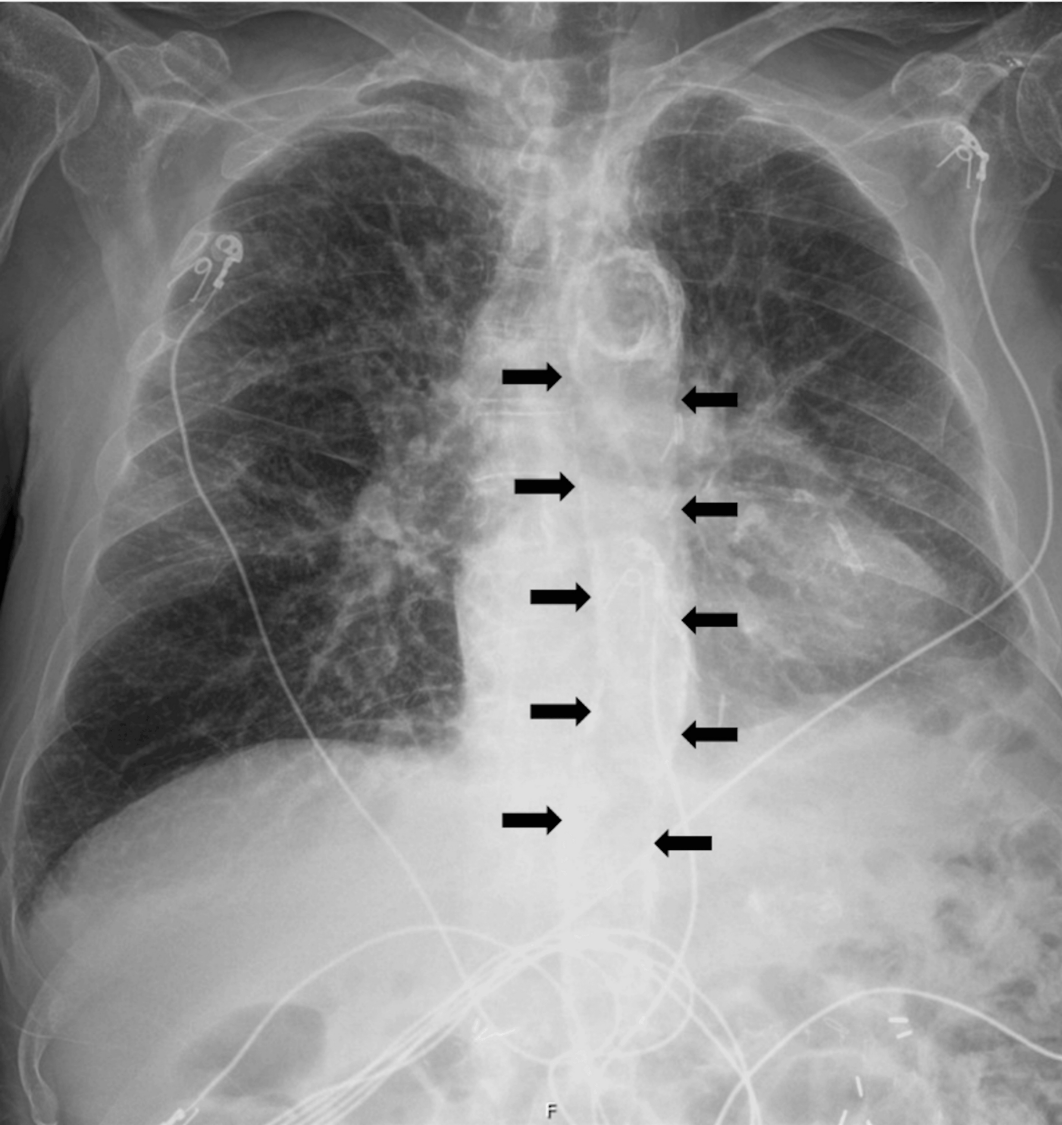
This image shows a posterior-anterior (PA) chest X-ray. There is extensive calcification of the aorta (black arrows).

**Figure 2:**
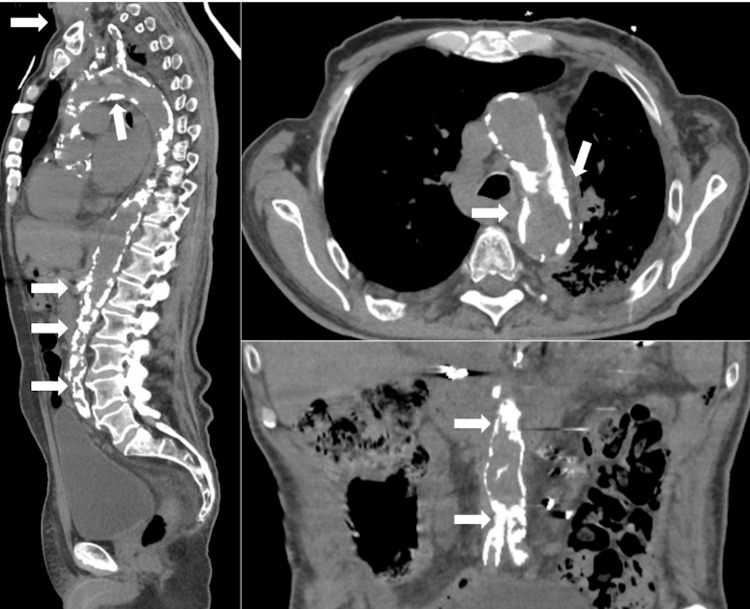
Selected views (sagittal left, axial top right, coronal bottom right) portraying extensive aortic calcification through the aortic arch down to the iliac arteries (white arrows).

One month later, the patient was referred to the hospital again from urgent care due to generalized weakness, ambulatory dysfunction, and hypotension. On initial assessment, the patient’s PBP was 79/37, and he was given multiple fluid boluses and ultimately was started on norepinephrine and broad-spectrum antibiotics for concerns of septic shock. Pertinent labs at the time were a lactate of 2.1 mmol/L (0.5-2.2 normal range), normal liver enzymes, and normal creatinine at 1 mg/dL (0.6-1.2 normal range). In addition, the patient had normal mental status and no other signs of organ dysfunction. CT head was done and showed no intracranial abnormalities. A chest x-ray and chest computed tomography with angiography were both performed showing extensive atheromatous calcifications in the arch of the aorta and the origins of the great vessels consistent with findings of porcelain aorta as well as no evidence of aortic dissection (Figures 1, 2). 

After several multidisciplinary discussions, it was decided to titrate vasopressors down as there were no signs of end-organ damage. An arterial line had been placed earlier but was discontinued as its readings matched those obtained from the blood pressure cuff. Non-invasive measurements were deemed acceptable while still accounting for the central-peripheral pressure gradient.

Subsequently, over the next few months, he was referred to the hospital a number of times from outpatient offices for low PBP. In addition, he had multiple intensive care unit admissions across a number of different hospitals. He underwent repeated imaging and diagnostic tests, and after extensive discussions, he opted for palliative care, focusing on symptom management rather than aggressive interventions. This included refraining from the use of vasopressors or intensive care unit admissions for recurrent episodes of asymptomatic hypotension, with the goal of minimizing invasive procedures and prioritizing quality of life, as long as there was no organ dysfunction. Extensive patient education was done to ensure an understanding of his condition and the limitations of non-invasive measurements.

## Discussion

The variation between CBP and PBP in patients with porcelain aorta is primarily driven by the loss of aortic compliance and stiffening of the aortic wall. Under normal conditions, CBP is slightly lower than PBP due to a phenomenon known as pulse pressure amplification. This is because the pressure wave increases at it travels through the arterial tree, increasing systolic pressure in peripheral arteries [3]. However, with aging and aortic calcification, increased arterial stiffness reduces elasticity, accelerating pulse wave velocity and causing reflected waves to return to the aorta during systole, leading to elevated central blood pressures and widened pulse pressures. Peripheral arteries, being more muscular and less elastic are less affected by calcification, resulting in lower PBP measurements relative to CBP in such conditions [4]. In our patient, this phenomenon was extreme, with a >100 mmHg difference between CBP and PBP, a degree of variation not previously reported [1]. This discrepancy complicates the clinical assessment of hypoperfusion, particularly in the setting of shock as peripheral measurements may underestimate true hemodynamics.

Inaccurate PBP readings can lead to mismanagement, such as admissions to the hospital, unnecessary fluid resuscitation, inappropriate use of vasopressors, and overuse of antibiotics. Another challenge in this population is determining the most reliable site for blood pressure measurement. Recent evidence suggests that CBP is a more accurate reflection of true hypoperfusion [5], but obtaining these measurements requires invasive procedures that expose patients to potential complications such as infections, bleeding, and vascular injury.

Management of porcelain aorta is primarily focused on medical optimization, as surgical options are limited [5]. This includes confirming the discrepancy between CBP and PBP, often using cardiac catheterization to accurately assess hemodynamics. Multi-disciplinary discussions are critical to establishing individualized blood pressure goals and ensuring organ function is carefully monitored to avoid harm. Alternative management strategies, such as using agents like Midodrine or Droxidopa to raise peripheral blood pressure, have been explored [6]. However, these approaches require aggressive outpatient monitoring and are likely suboptimal, given that CBP more accurately reflects true perfusion. Patient education plays a central role in managing this condition. It is essential to ensure patients understand their diagnosis, the implications of their blood pressure measurements, and the importance of communicating their condition to healthcare providers during future encounters. Our patient tolerated lower peripheral blood pressures without evidence of end-organ damage and was safely discharged multiple times, emphasizing the importance of individualized care.

Furthermore, porcelain aorta has traditionally been recognized as a significant barrier in aortic valve replacement and other cardiac surgeries, particularly in patients with severe aortic stenosis. The increased CBP in these patients further complicates surgical procedures due to the stiffness and fragility of the calcified aorta. A penetrating calcified aorta makes aortic cannulation for bypass and cross-clamping particularly difficult, increasing the risk of cracking the aortic wall, which can lead to stroke, embolization, or bleeding [7]. Advanced surgical strategies, including performing the procedure off-pump, using clampless devices, or inducing hypothermia, are often employed to reduce the risk of injury to the aorta [8]. Additionally, alternative cannulation sites such as the femoral or axillary arteries are utilized to minimize manipulation of the aorta, thus lowering the risk of complications [8].

Our case is unique as the patient presented outside of surgery. Management focused on not only conservative care with non-invasive measures but also patient education. Multidisciplinary discussions were essential to balance blood pressure control while avoiding harm. This highlights the importance of individualized care for patients with porcelain aorta, in the non-surgical setting.

## Conclusions

This case reflects the importance of individualized management strategies, especially the careful interpretation of hemodynamic data in the context of an overall clinical status. The presence of porcelain aorta may serve as a marker for advanced vascular disease, highlighting the need for a multidisciplinary approach to optimize care and quality of life in these patients.
